# Machine Learning-Guided Screening and Molecular Docking for Proposing Naturally Derived Drug Candidates Against MERS-CoV 3CL Protease

**DOI:** 10.3390/ijms26073047

**Published:** 2025-03-26

**Authors:** Mebarka Ouassaf, Radhia Mazri, Shafi Ullah Khan, Kannan R. R. Rengasamy, Bader Y. Alhatlani

**Affiliations:** 1 Group of Computational and Medicinal Chemistry, LMCE Laboratory, University of Biskra, Biskra 07000, Algeria; radhia.mazri@univ-biskra.dz; 2 Inserm U1086 ANTICIPE (Interdisciplinary Research Unit for Cancer Prevention and Treatment), Normandie Univ, Université de Caen Normandie, 14076 Caen, France; shafiullahpharmd@gmail.com; 3 Comprehensive Cancer Center François Baclesse, UNICANCER, 14076 Caen, France; 4 Laboratory of Natural Products and Medicinal Chemistry (LNPMC), Department of Pharmacology, Saveetha Dental College and Hospitals, Saveetha Institute of Medical and Technical Sciences (SIMATS), Thandalam, Chennai 602105, India; rengasamy@iceir.net; 5 Centre of Excellence for Pharmaceutical Sciences, North-West University, Potchefstroom 2520, South Africa; 6 Unit of Scientific Research, Applied College, Qassim University, Buraydah 52571, Saudi Arabia

**Keywords:** machine learning, MERS-CoV, random forest, molecular docking, molecular dynamics simulations

## Abstract

In this study, we utilized machine learning techniques to identify potential inhibitors of the MERS-CoV 3CL protease. Among the models evaluated, the Random Forest (RF) algorithm exhibited the highest predictive performance, achieving an accuracy of 0.97, an ROC-AUC score of 0.98, and an F1-score of 0.98. Following model validation, we applied it to a dataset of 14,194 naturally occurring compounds from PubChem. The top-ranked compounds were subsequently subjected to molecular docking, which identified Perenniporide B, Phellifuropyranone A, and Terrestrol G as the most promising candidates, with binding energies of −9.17, −9.08, and −8.71 kcal/mol, respectively. These compounds formed strong interactions with key catalytic residues, suggesting significant inhibitory potential against the viral protease. Furthermore, molecular dynamics simulations confirmed their stability within the active site, reinforcing their viability as antiviral agents. This study demonstrates the effectiveness of integrating machine learning with molecular modeling to accelerate the discovery of therapeutic candidates against emerging viral threats.

## 1. Introduction

Middle East respiratory syndrome coronavirus (MERS-CoV) is a major zoonotic pathogen with a high mortality rate, estimated at approximately 35% [1]. Primarily transmitted from camels to humans, the virus has caused significant epidemics across the Middle East, Asia, and North America. Human-to-human transmission, particularly in healthcare settings, has also been documented [2]. One of MERS-CoV’s key immune evasion strategies involves its non-structural proteins, notably nsp5, which inhibits type I interferon (IFN) production by preventing the nuclear translocation of IRF3, a critical regulator of antiviral signaling [3,4,5]. Despite extensive research efforts, including in silico approaches targeting viral proteins such as the M protein, controlling MERS-CoV remains a challenge due to the absence of effective antiviral treatments [6]. Addressing this gap is crucial for improving public health strategies and mitigating the global impact of MERS-CoV.

The MERS-CoV 3-chymotrypsin-like protease (3CLpro) is a key therapeutic target due to its essential role in viral replication and maturation [7]. This protease is responsible for cleaving viral polyproteins, a process that depends on its dimerization and the unique architecture of its active site [8]. The development of 3CLpro inhibitors is therefore a priority, particularly in the absence of approved antiviral therapies against MERS-CoV.

Traditional drug discovery approaches, such as high-throughput screening (HTS) and experimental enzymatic assays, often require significant financial and time investments, limiting their applicability in pandemic preparedness [9]. Computational drug discovery has revolutionized this field by enabling in silico screening of large compound libraries through molecular docking, molecular dynamics (MD) simulations, and quantitative structure–activity relationship (QSAR) modeling [10]. These methods provide valuable insights into ligand–protein interactions, binding affinities, and conformational stability, facilitating the identification of promising lead compounds.

Recent advancements in machine learning (ML) have further enhanced computational drug discovery by addressing the limitations of conventional screening methods. ML algorithms can analyze vast chemical and biological datasets, predict compound bioactivity, and refine docking and QSAR models for improved accuracy [11,12]. Studies have demonstrated that ML-assisted virtual screening can significantly reduce false positives, enhance hit identification, and optimize lead selection compared to traditional docking-based approaches alone [13,14]. Therefore, integrating ML with molecular simulations presents a promising strategy for identifying novel MERS-CoV 3CLpro inhibitors more efficiently and reliably.

Several computational studies have explored potential inhibitors against MERS-CoV 3CLpro, primarily relying on molecular docking and virtual screening approaches [15,16]. However, few studies have combined ML-driven predictive models with molecular simulations to improve screening accuracy and prioritize lead compounds based on both binding affinity and pharmacokinetic properties. This study aims to bridge this gap by employing a hybrid ML-assisted virtual screening approach, followed by molecular docking, MD simulations, and ADMET (absorption, distribution, metabolism, excretion, and toxicity) analysis to identify potent natural inhibitors of MERS-CoV 3CLpro. Natural compounds have demonstrated antiviral potential against various coronaviruses, offering structural diversity and bioactive properties that enhance drug discovery pipelines [17].

In this study, we aim to identify potential inhibitors of MERS-CoV 3CLpro using machine learning techniques to develop a predictive model based on compounds with demonstrated antiviral activity, despite the limited availability of relevant datasets. Following ML-based screening, we have employed molecular docking, ADMET (absorption, distribution, metabolism, excretion, and toxicity) analysis, and molecular dynamics simulations to further evaluate promising candidates. This integrative approach seeks to propose naturally occurring compounds as potential leads for novel antiviral therapies against MERS-CoV.

## 2. Results

### 2.1. Model Performance

To identify potential inhibitors of the MERS-CoV 3CL protease, we developed multiple machine learning models using various techniques, as detailed in Section 2.1. The performance and outcomes of these models are summarized in Table 1.

Among the tested approaches, the Random Forest (RF) model demonstrated the highest predictive performance [18], achieving an accuracy of 0.97, an ROC-AUC score of 0.98, and an F1-score of 0.98. These results highlight its robustness and reliability for this classification task. In contrast, alternative models such as Logistic Regression (LR) and Support Vector Machine (SVM), while performing adequately (accuracy between 0.78 and 0.81, F1-scores ranging from 0.77 to 0.82), did not reach the level of precision and generalizability achieved by RF.

The K-Nearest Neighbors (KNN) model, although useful in similar classification tasks, exhibited notable limitations in this study. It recorded a lower F1-score (0.68) and a macro-average score of 0.64, indicating potential difficulties in handling class imbalances and generalizing effectively.

Given its superior performance in capturing complex relationships within the data, the Random Forest model was selected as the optimal predictive tool for this study. Its high accuracy, robustness, and ability to effectively differentiate active and inactive compounds make it the most reliable choice for screening potential MERS-CoV 3CL protease inhibitors.

### 2.2. Validation and Visualization of Model Performance

**Figure 1:**
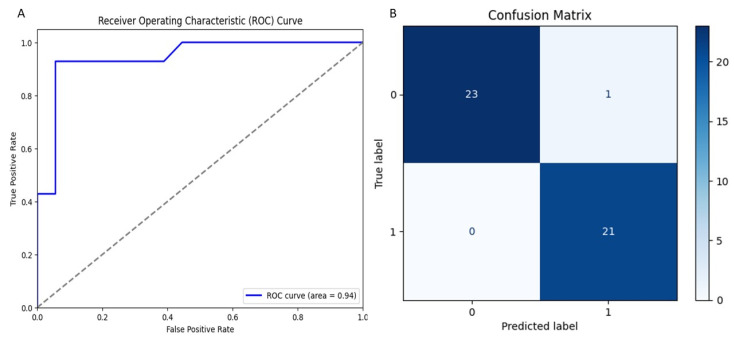
Evaluation of the Random Forest model: (**A**) ROC curve and (**B**) confusion matrix analysis.

After selecting Random Forest as the top-performing model, we conducted thorough validation to assess its reliability. The receiver operating characteristic (ROC) curve (Figure 1A) showed an impressive area under the curve (AUC) of 0.94, demonstrating the model’s strong ability to differentiate between active and inactive compounds. The dotted diagonal line represents the performance of a random classifier (AUC = 0.5), indicating no discrimination ability. Any classifier with an AUC greater than 0.5 performs better than random guessing. The closer the AUC is to 1.0, the better the model’s predictive performance. This balance between sensitivity and specificity is crucial for making accurate classification decisions. Additionally, the confusion matrix (Figure 1B) reinforced the model’s robustness, with 23 correct predictions and only one false positive for class 0, alongside 21 correct predictions and no false negatives for class 1. Notably, the model accurately identified all active compounds without any misclassification, making it a highly effective tool for screening potential drug candidates with pharmaceutical significance.

**Figure 2:**
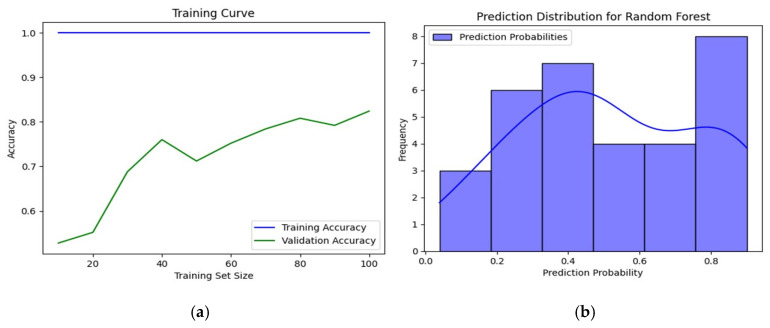
Evaluation of the Random Forest model: (**a**) training curve and (**b**) prediction distribution histogram.

The training accuracy graph (Figure 2a) illustrates a positive trend in model performance, where the training accuracy remains stable at 1.0, while the validation accuracy progressively increases to approximately 0.85 as the training dataset expands. This trend reflects the model’s ability to improve generalization, ensuring robust performance on unseen data.

Additionally, the distribution analysis of prediction probabilities (Figure 2b) reveals a bimodal pattern, with frequency peaks in the intervals [0.3–0.4] and [0.8–0.9]. This suggests that the model assigns higher confidence to predictions within these ranges. The superimposed curve further indicates a balanced management of uncertainty levels, reinforcing the flexibility and reliability of the model in its decision-making process.

These combined analyses confirm that Random Forest is a powerful and robust tool, ideally suited for prioritizing promising compounds as candidates for therapeutic development.

After validating the model to ensure its accuracy, we applied it on a large scale to screen a broader set of naturally occurring compounds for potential MERS-CoV 3CL protease inhibitors. This approach aims to accelerate the discovery of active compounds that could serve as a foundation for the development of new antiviral treatments.

The dataset for this study was sourced from PubChem, comprising a total of 14,194 naturally occurring compounds. Using the Random Forest model, we analyzed these compounds to predict their potential inhibitory activity against the MERS-CoV 3CL protease. Among them, 1232 compounds were identified as active, representing approximately 8.67% of the dataset. This substantial proportion of promising compounds underscores the potential of our screening approach in identifying biologically relevant candidates.

**Figure 3:**
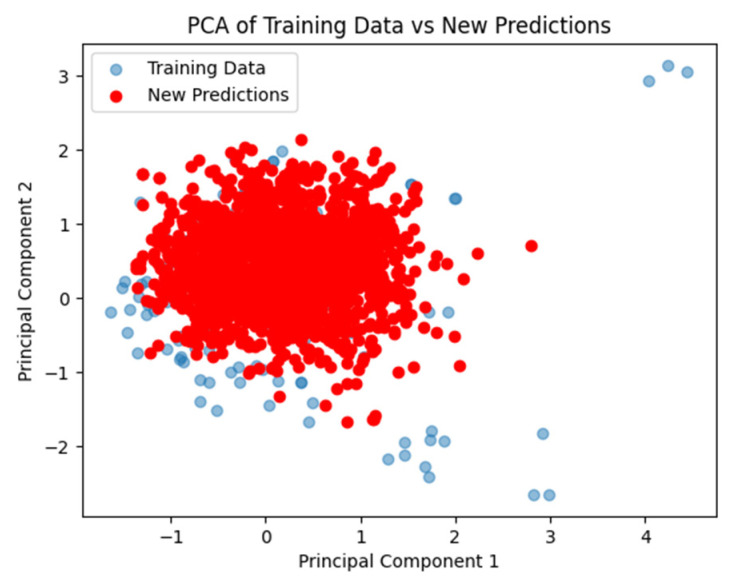
PCA of training data vs. new predictions: Random Forest model.

To further evaluate the reliability of our predictions, we conducted a principal component analysis (PCA), as illustrated in Figure 3 (PCA of training data vs. new predictions). This visualization reduces the high-dimensional molecular fingerprint data to a two-dimensional space, facilitating a direct comparison between the training data (blue dots) and the new predictions (red dots). The high proximity and overlap between these two datasets indicate that the majority of new predictions fall within the chemical space defined by the training data, confirming the model’s reliability and applicability. This visualization highlights the robustness of the model, reinforcing confidence in its ability to generate accurate predictions within its domain of applicability. 

### 2.3. Docking Study and Binding Interaction Analysis of Compounds with MERS-CoV 3CLpro

After using the Random Forest model to predict compounds with potential efficacy, we will conduct molecular docking on the 1232 selected compounds (File S1). Molecular docking is a key technique in in silico drug design, allowing the assessment of binding affinity and molecular interactions between potential compounds and the MERS-CoV 3CL protease. This step allows for the prioritization of the most promising inhibitors by identifying compounds with strong binding affinities and favorable interaction profiles. The results of this analysis will serve as the foundation for further computational validation studies, advancing the identification of potential antiviral candidates against MERS-CoV.

We initiated a molecular docking study to evaluate the binding potential of selected compounds from the Random Forest model within the active site of the MERS-CoV protease. Initially, docking was performed using the standard precision (SP) mode, yielding 520 compounds with docking scores lower than −6 kcal/mol. A more rigorous extra precision (XP) docking was then conducted on these compounds, identifying 94 molecules with superior binding affinities compared to the reference inhibitor (<= −7.34 kcal/mol) (Table S2). To refine the selection, we applied additional filtering criteria based on toxicity and absorption properties, ultimately identifying the three most promising compounds with known sources: Perenniporide B, Phellifuropyranone A, and Terrestrol G.

The compounds Perenniporide B, Phellifuropyranone A, and Terrestrol G exhibited strong binding affinities for the MERS-CoV 3CL protease, with binding energies of −9.17, −9.08, and −8.71 kcal/mol, respectively (Table 2 and Table 3). These values indicate a significant inhibitory potential, suggesting that these compounds may effectively disrupt the catalytic activity of the viral enzyme, thereby hindering viral replication in host cells.

**Figure 4:**
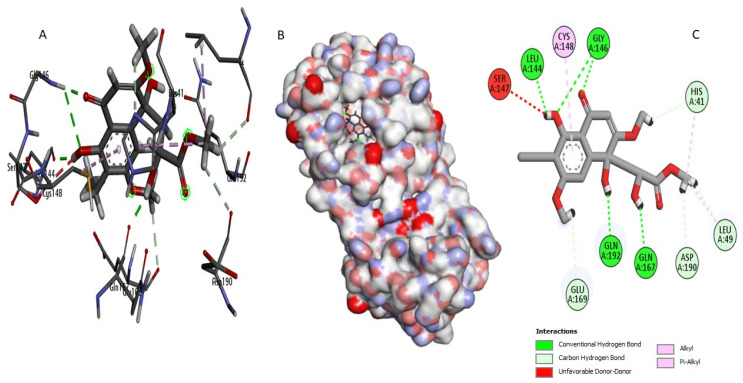
Molecular interaction analysis of the MERS-CoV 3CLpro complex with Perenniporide B: (**B**) surface view, (**A**) 3D structure, and (**C**) interaction map.

Perenniporide B (CID60199564) established strong interactions within the enzyme’s active site (Figure 4). It formed short hydrogen bonds (1.7–2.9 Å) with critical catalytic residues, including His41, which is directly involved in the proteolytic cleavage mechanism, and Asp190, which plays a key role in stabilizing the enzyme’s tertiary structure [19]. Additionally, Perenniporide B exhibited hydrophobic interactions with Leu144 and Leu49, along with a moderate sulfur interaction with Cys148 (5.1 Å), suggesting strong anchoring within the active pocket that may prevent substrate binding and enzymatic function.

**Figure 5:**
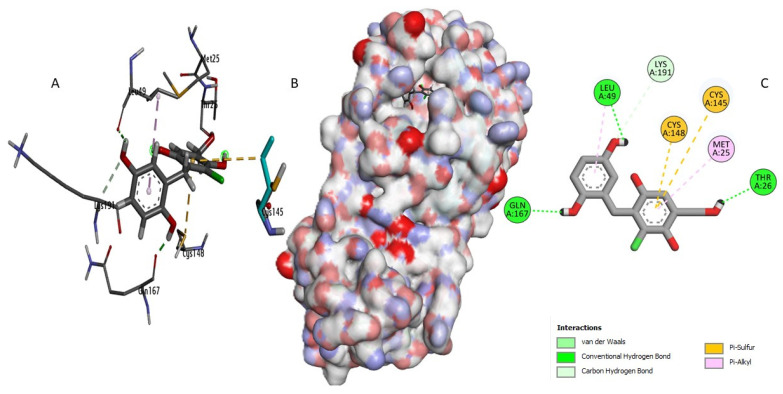
Molecular interaction analysis of the MERS-CoV 3CLpro complex with Terrestrol G: (**B**) surface view, (**A**) 3D structure, and (**C**) interaction map.

Terrestrol G (CID24761035) exhibited a diverse interaction profile (Figure 5), forming two sulfur interactions (4.3 Å and 5.7 Å) with the catalytic residue Cys145/Cys148, which are essential for enzymatic activity [20,21]. Additionally, it established four hydrogen bonds, including a short hydrogen bond (1.7 Å) with Thr26, a key residue for substrate recognition, and engaged in two hydrophobic interactions that further stabilized its binding. These multiple interactions suggest that Terrestrol G may effectively anchor within the active site and interfere with enzymatic function, making it a promising candidate for inhibition.

**Figure 6:**
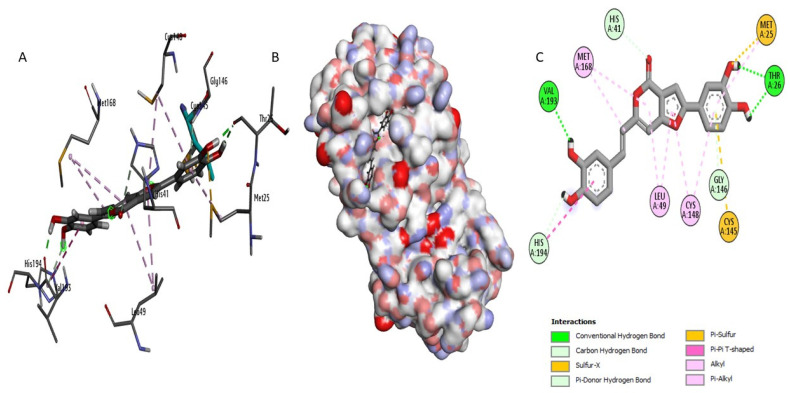
Molecular interaction analysis of the MERS-CoV 3CLpro complex with Phellifuropyranone A: (**B**) surface view, (**A**) 3D structure, and (**C**) interaction map.

On the other hand, Phellifuropyranone A (CID24770409) showed interaction with His41 but had limitations in its inhibitory potential (Figure 6). Its sulfur bonds with Cys148 (4.5 Å) and Met25 (3 Å) were relatively long, reducing their effectiveness in stabilizing the compound within the active site. Additionally, the rigid structural framework of Phellifuropyranone A might limit its adaptability, further compromising its binding efficiency.

To evaluate their inhibitory potential, the newly identified compounds were compared with the reference inhibitor AW4 (CID137348956) (Figure S1). Docking analysis revealed that AW4 formed hydrogen bonds with Glu169 (1.88 Å) and Tyr54 but lacked direct interactions with Cys145, a key residue for competitive inhibition. Its hydrophobic interactions with Leu49 and Val193 were also less specific than those observed for Perenniporide B and Terrestrol G, potentially explaining its lower binding affinity. In contrast, Phellifuropyranone A and Terrestrol G exhibited superior interaction profiles, engaging His41 through hydrogen bonding and Cys145/Cys148 via sulfur interactions, suggesting their strong potential as synergistic enzyme inhibitors. Perenniporide B demonstrated even more extensive hydrogen bonding, further reinforcing its inhibitory potential. The minimal engagement of AW4 with key catalytic residues highlights the stronger and more stable binding achieved by our identified compounds.

### 2.4. Evaluation of Pharmacological Profiles and ADMET Characteristics

Following the docking studies and the analysis of binding interactions at the active site, we conducted a comprehensive evaluation of the physicochemical properties, pharmacokinetics, and toxicity profiles of the selected compounds. These assessments are essential for determining their drug-likeness, bioavailability, and safety, which are critical factors in the early stages of drug development.

To ensure a thorough comparison, the selected compounds were systematically evaluated alongside the reference inhibitor AW4. This comparison allowed us to assess how the newly identified inhibitors perform relative to a known active compound in terms of absorption, distribution, metabolism, excretion (ADME), and toxicity risks. By integrating these analyses, we aim to identify the most promising candidates for further preclinical development while minimizing potential toxicity concerns or pharmacokinetic limitations.

#### 2.4.1. Prediction of Compound Physicochemical Properties

The analysis of the physicochemical properties (Table 4) of the compounds highlights characteristics that favor their potential to inhibit MERS-CoV 3CLpro. Perenniporide B and Terrestrol G are distinguished by their moderate molecular weight (366.13 and 296.05 Da, respectively) and low flexibility, factors that may favor good oral bioavailability. Phellifuropyranone A, with a more marked hydrophobicity (Log P = 3.562), could effectively interact with the enzymatic target, although its solubility profile (Log S = −4.103) may require optimization. In comparison, the reference compound AW4 (533.16 Da) has a higher flexibility (nRot = 15) and a larger accessible polar surface area (TPSA = 171.13 Å^2^), which may favor specific interactions with some polar residues of the enzyme, but could also limit its membrane permeability. Conversely, Perenniporide B and Terrestrol G, with respective TPSAs of 122.52 and 101.15 Å^2^, offer a good balance between solubility and membrane diffusion, thus optimizing their pharmacokinetic potential.

These results show that Perenniporide B and Terrestrol G have a favorable profile in terms of stability and molecular interactions, while Phellifuropyranone A could benefit from adjustments to improve its solubility. Comparison with AW4 highlights the strengths and areas for optimization of the novel compounds, underlining their potential as MERS-CoV 3CLpro inhibitors.

#### 2.4.2. Evaluation of Medicinal Chemistry and Drug-Likeness Properties

The analysis of medicinal chemistry and drug-likeness properties (Table 5) highlights the distinct characteristics of the selected compounds, which influence their potential as MERS-CoV 3CL protease inhibitors.

From a medicinal chemistry perspective, all compounds comply with Lipinski’s Rule of Five and Pfizer’s guidelines, suggesting potential oral bioavailability. Perenniporide B and Terrestrol G demonstrate high quantitative estimate of drug-likeness (QED) scores (0.632 and 0.559, respectively), reflecting balanced pharmacological profiles. The reference compound AW4 has a QED (quantitative estimate of drug-likeness) of 0.25, indicating a more limited development potential compared to the other compounds studied. Its SAscore (synthetic accessibility score) of 3.988 is relatively high, suggesting a potentially more complex synthesis. Although AW4 meets the Pfizer rule, it does not meet the Lipinski rule or the Golden Triangle criteria, which may indicate challenges in terms of bioavailability and pharmaceutical development. In comparison, Perenniporide B, Phellifuropyranone A, and Terrestrol G meet all the evaluation rules, highlighting their better balance between potential efficacy and favorable pharmacokinetic properties.

The absorption and distribution profiles (Table 6) of the selected compounds reveal key pharmacokinetic characteristics that influence their potential as MERS-CoV 3CL protease inhibitors. The human intestinal absorption (HIA%) values indicate satisfactory absorption for all three compounds: Perenniporide B (65.62%), Phellifuropyranone A (63.39%), and Terrestrol G (67.33%). However, their low Caco-2 permeability values (ranging from −4.922 to −5.168) suggest limited passive diffusion, which may necessitate the use of advanced drug delivery strategies, such as nanoparticles or prodrug formulations, to enhance their bioavailability.

The plasma protein binding (PPB%) values indicate a strong binding affinity to serum proteins, particularly for Phellifuropyranone A (96.695%) and Terrestrol G (95.148%). This high protein-binding tendency could reduce their active free fraction, potentially impacting their therapeutic efficacy by limiting the bioavailable drug concentration in plasma.

The volume of distribution (Vd) provides further insights into tissue distribution patterns. Perenniporide B (1.259 L/kg) demonstrates a higher volume of distribution, indicating extensive tissue penetration and potential intracellular accumulation. In contrast, Phellifuropyranone A (0.355 L/kg) appears to be primarily confined to plasma, suggesting limited distribution beyond the bloodstream. These differences in distribution behavior may have implications for drug action duration and target tissue accessibility, influencing their suitability for further therapeutic development.

The metabolism and excretion profiles (Table 7) of the compounds reveal important considerations for their pharmacokinetic behavior and safety. Phellifuropyranone A stands out due to its strong inhibition of CYP1A2 (0.934) and CYP3A4 (0.498) enzymes, which could significantly increase the risk of drug–drug interactions. This inhibition could alter the metabolism of other drugs, leading to altered drug levels and potential side effects.

In contrast, Perenniporide B and Terrestrol G exhibit moderate cytochrome P450 inhibition, suggesting a more predictable metabolism and potentially fewer drug interaction risks.

Regarding elimination, Terrestrol G shows a high clearance rate (13.677 mL/min/kg) and a short half-life (0.966 h), indicating rapid drug elimination. While this suggests efficient clearance from the body, it may also necessitate frequent dosing to maintain therapeutic levels. On the other hand, AW4 has a very low clearance (2.121 mL/min/kg), suggesting that it could accumulate in the body, which may require increased monitoring to prevent potential toxicity and ensure safe use.

These insights into metabolism and excretion provide a comprehensive understanding of the pharmacokinetic profiles of the compounds, helping to guide dosing strategies and identify potential safety concerns during further development.

#### 2.4.3. Toxicity Profile and Pharmacological Implications

The toxicity profiles of the compounds studied are generally favorable, showing no significant signs of mutagenicity, cytotoxicity, or hepatotoxicity (Table 8 and Table 9). However, certain toxicological characteristics warrant particular attention during further development.

Respiratory toxicity assessments for the investigated compounds, including Perenniporide B, were conducted using ADMELab, a QSAR-based predictive tool that models cytotoxic interactions with respiratory cell targets. Perenniporide B exhibited a score of **0.669**, which, while higher than the other studied compounds, remains within an intermediate range compared to clinically approved antivirals assessed using the same ADMELab protocol (see Methods and Table S3 for comparative data).

Phellifuropyranone A and Terrestrol G display high skin sensitization scores (0.951 and 0.952, respectively), indicating a potential risk of T-cell-mediated allergic reactions. This is particularly important for both topical and systemic administrations, as their metabolites could trigger dermatitis in sensitive individuals.

Both Terrestrol G and AW4 exhibit high LD50 values (2500 and 3000 mg/kg), suggesting low acute toxicity and indicating a wide therapeutic index. On the other hand, Perenniporide B has a relatively low LD50 (220 mg/kg), implying a higher toxic risk at high doses, thus requiring rigorous dose monitoring in preclinical studies to ensure safe usage.

These findings underscore the need for careful route of administration and dose adjustments to mitigate the specific risks of each compound. Nevertheless, the compounds generally demonstrate no major organ toxicity, emphasizing their therapeutic potential when handled properly.

### 2.5. Molecular Dynamics Simulation Analysis

Molecular dynamics (MD) simulations offer valuable insights into the stability and dynamic behavior of ligand–protein complexes over time. In this study, MD simulations were employed to evaluate the interactions of natural compounds and the reference compound with the MERS-CoV protease. The analysis focused on key parameters, such as RMSD (Root Mean Square Deviation), RMSF (Root Mean Square Fluctuation), hydrogen bonding, and interaction stability, to assess both the binding affinity and the structural integrity of the complexes.

RMSD analysis is critical for evaluating the stability of protein–small molecule interactions during molecular simulations. Low RMSD values and limited fluctuations indicate a stable interaction, while high variations or abrupt jumps suggest significant structural changes or low binding affinity [22].

**Figure 7:**
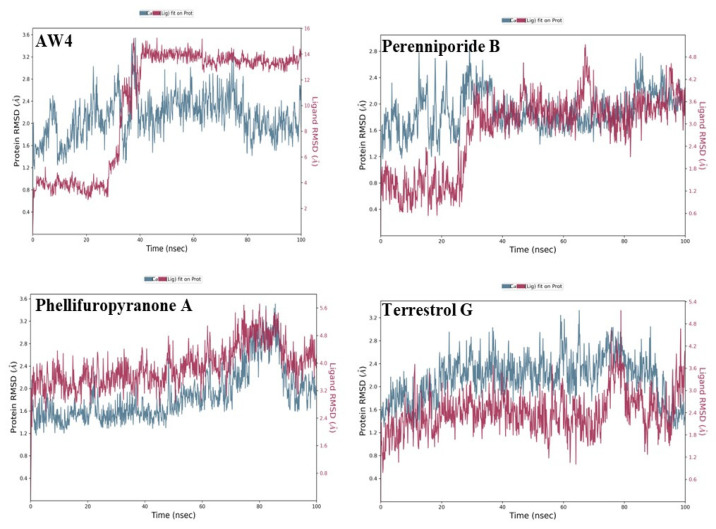
RMSD curves of natural compounds and the reference compound AW4 with the MERS-CoV Protease.

In this study, RMSD analysis (Figure 7) reveals that Phellifuropyranone A and Terrestrol G exhibit remarkable stability in their interaction with the main protease of MERS-CoV. The protein RMSDs for these compounds range from 1.4 to 2.2 Å and 1.6 to 2.4 Å, while the ligand RMSDs range from 1.0 to 5.6 Å and 1.2 to 4.8 Å, respectively. These values demonstrate their exceptional ability to maintain stable interactions with the target enzyme, suggesting a high binding affinity and potential for long-lasting inhibition.

Similarly, Perenniporide B also shows significant stability, with protein RMSD ranging from 1.8 to 2.6 Å and ligand RMSD from 1.2 to 4.2 Å. This suggests that Perenniporide B exhibits good retention within the protease’s active site, indicating a favorable dynamic profile for effective inhibition.

These results underscore the ability of Phellifuropyranone A, Terrestrol G, and Perenniporide B to act as effective inhibitors of MERS-CoV’s main protease, demonstrating favorable dynamic stability that could support prolonged antiviral action.

In comparison, the reference compound AW4 exhibits higher variability, with protein RMSD ranging from 2.5 to 3.2 Å and ligand RMSD fluctuating between 6 and 14 Å. A notable increase in these values after 40 nanoseconds suggests that AW4 undergoes more complex structural adjustments, possibly indicating a lower affinity for the active site or less stable binding interactions. This contrast highlights the superior stability of Phellifuropyranone A, Terrestrol G, and Perenniporide B, which show more consistent and favorable RMSD profiles, suggesting their potential for more effective and stable interactions with the MERS-CoV protease than the reference compound.

**Figure 8:**
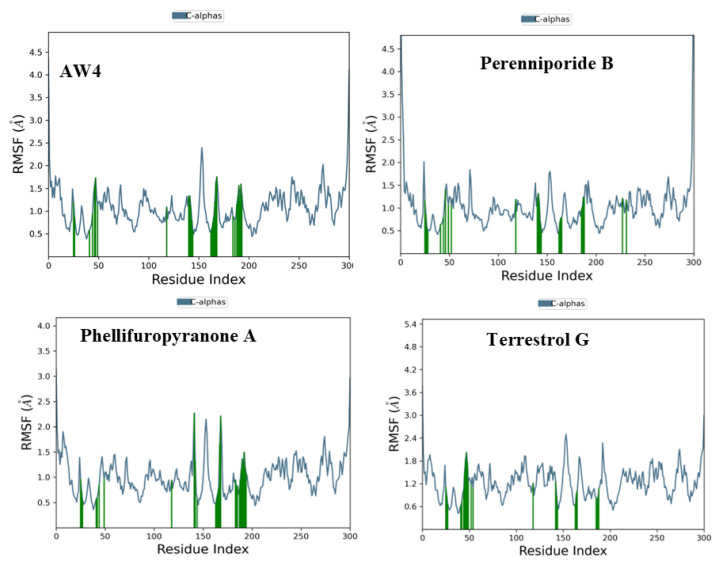
RMSF curves of natural compounds and the reference compound AW4 with the MERS-CoV Protease.

Furthermore, Root Mean Square Fluctuation (RMSF) analysis provides insights into the flexibility and stability of the complexes formed between the protease and various ligands [23]. For AW4, moderate stability is observed, with fluctuations ranging from 0.5 to 3.5 Å. A prominent peak in RMSF (Figure 8) is seen around the C-terminus (280–300), indicating some regional flexibility in that area. This increased flexibility at specific regions may contribute to the compound’s reduced binding stability, especially in comparison to the more stable interactions observed with the natural compounds.

The enhanced stability and lower fluctuations observed for Phellifuropyranone A, Terrestrol G, and Perenniporide B further emphasize their promising profiles for development into potent therapeutics targeting the MERS-CoV protease. Their more stable interactions suggest a potentially greater efficacy for long-term inhibition of viral activity compared to AW4.

The proposed inhibitors exhibit competitive or even improved fluctuation profiles compared to AW4, further supporting their potential as strong candidates for therapeutic development. Phellifuropyranone A, with fluctuations ranging from 0.5 to 4.0 Å, demonstrates excellent stability, making it a viable competitor to the reference compound in terms of interaction with the MERS-CoV protease. On the other hand, Perenniporide B and Terrestrol G display slightly more pronounced fluctuations, ranging from 0.5 to 4.5 Å and 0.5 to 5.4 Å, respectively. These variations, while greater than those of Phellifuropyranone A, can be interpreted as evidence of the compounds’ adaptability and dynamic adjustment at the protein active site, which might be beneficial for efficient inhibition.

In comparison to AW4, the three studied inhibitors demonstrate a robust interaction with the MERS-CoV protease, with fluctuations that suggest strategic flexibility in their binding. These variations are not necessarily instabilities but could reflect an ability to adjust and optimize their interactions at the active site, potentially leading to more efficient inhibition of the viral enzyme. The dynamic nature of these compounds may enhance their occupancy of the active site, allowing them to compete effectively with AW4.

Based on these findings, Phellifuropyranone A, Perenniporide B, and Terrestrol G emerge as promising candidates. Their favorable fluctuation profiles highlight their potential for developing potent and flexible inhibitors of the MERS-CoV protease, warranting further investigation into their inhibitory efficacy and potential structural optimization to enhance their drug-like properties.

**Figure 9:**
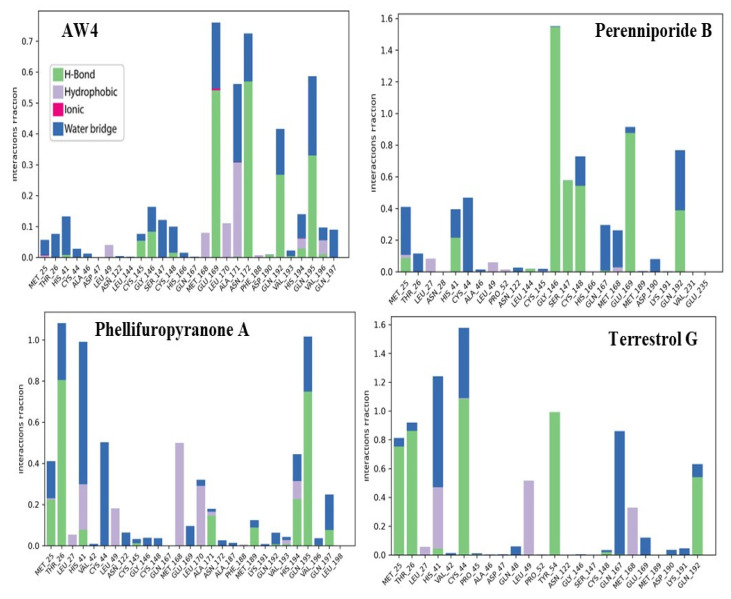
Histogram of interaction frequencies of natural compounds and the reference compound with the MERS-CoV protease.

The analysis of interactions between the compounds and the MERS-CoV main protease reveals notable differences in how each compound engages with the critical residues of the active site, which could impact their inhibitory potential (Figure 9).

Phellipuropyranone A and Terrestrol G establish persistent and stable contacts with several key residues in the active site, including Met 25, Thr 26, Ile 27, His 41, and Glu 166. Their interaction fractions range from 1.0 to 1.4, indicating continuous and stable interactions. These compounds show a strong presence of hydrogen bonds (green) and hydrophobic interactions (blue), reflecting a robust binding to the active site. This pattern of interactions suggests that Phellifuropyranone A and Terrestrol G are likely to exert strong inhibitory effects by stabilizing their positions within the protease, potentially preventing the enzyme from performing its catalytic functions.

In contrast, Perenniporide B demonstrates a dominant interaction with Cys 145 and His 164, two essential residues for the catalytic activity of the protease [24]. The interaction fractions for these residues reach 1.5, indicating a particularly strong binding to these critical sites. This suggests that Perenniporide B may selectively target and inhibit the enzyme by directly interfering with its catalytic machinery. The preferential binding to Cys 145 and His 164 could lead to more efficient inhibition of the protease, as these residues play central roles in the enzyme’s activity.

These findings underline the importance of residue specificity in determining the strength and effectiveness of the compounds as inhibitors of the MERS-CoV protease. Phellifuropyranone A and Terrestrol G benefit from broader interactions with key residues, while Perenniporide B takes advantage of more targeted interactions with residues critical to the protease’s function, further emphasizing the potential of these compounds for inhibitory efficacy.

The reference compound AW4 establishes relatively diversified interactions, but these interactions are more variable in intensity. While Glu 166 and Asp 187 are involved in the binding, the overall strength and specificity of these interactions are weaker compared to the other compounds. In contrast, Phellifuropyranone A, Terrestrol G, and Perenniporide B form more intense and specific interactions with the protease, particularly with critical residues involved in the enzyme’s catalytic function. Their long-lasting bonds with key residues like His 41, Cys 145, His 164, Met 25, and Glu 166 suggest a much stronger affinity for the protease, making them promising candidates for further development as effective antiviral agents.

**Figure 10:**
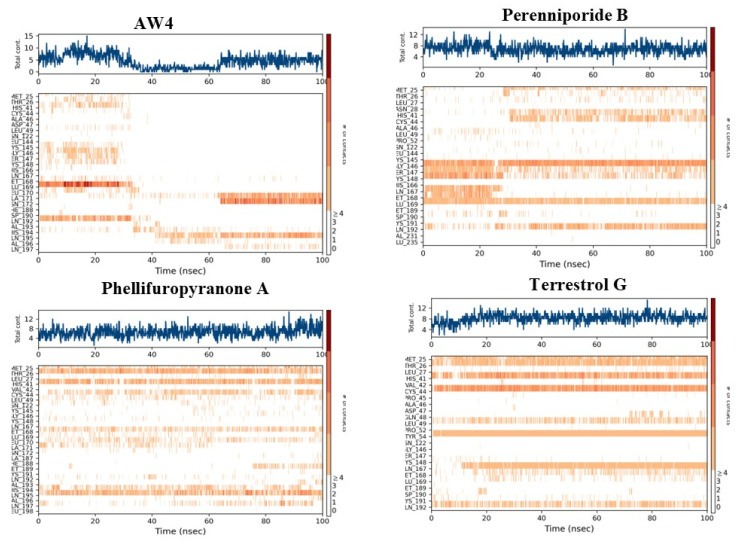
Heatmap of binding interaction profiles of natural compounds and the reference compound with the MERS-CoV protease.

Further supporting these findings, the heatmap analysis of the interactions during a 100 ns simulation provides a dynamic view of the stability and persistence of these interactions over time (Figure 10). The blue graph represents the variations in total cost over the simulation time. The reference compound AW4 exhibits larger fluctuations compared to the other compounds (Phellifuropyranone A, Terrestrol G, and Perenniporide B), which may indicate a different dynamic stability. In contrast, the three other compounds appear more stable with fewer fluctuations, possibly reflecting a more stable interaction with the biological target.

The reference compound AW4 shows dispersed and intermittent interactions, which suggests a lower stability and reduced binding affinity for the active site. This variability could indicate that AW4’s binding may be less consistent, and it might experience continuous reorganization within the binding site, thus diminishing its ability to effectively inhibit the protease.

On the other hand, Perenniporide B demonstrates more pronounced and prolonged interactions with critical residues such as His 41, Cys 145, His 164, and Glu 166. These residues are known to play crucial roles in the enzyme’s catalytic mechanism, and their sustained interaction with Perenniporide B suggests effective competitive inhibition, thereby preventing the natural substrate from binding and inhibiting the enzyme’s function.

Similarly, Phellifuropyranone A and Terrestrol G exhibit persistent interactions with key residues like Met 25, Thr 26, His 41, Glu 166, and Asp 187, enhancing the compounds’ ability to disrupt the protease’s enzymatic function.

When comparing the interaction fraction histograms of the compounds, a clear consistency emerges: AW4 exhibits unstable and less specific binding, while Phellifuropyranone A, Terrestrol G, and Perenniporide B demonstrate strong, stable, and continuous interactions with critical residues. This consistent and stable binding positions the latter three compounds as superior inhibitors, making them ideal candidates for further development. The findings emphasize the importance of interaction stability and affinity in selecting the most effective candidates for therapeutic applications targeting the MERS-CoV protease.

## 3. Discussion

This study integrates machine learning, molecular modeling, and pharmacokinetic analysis to identify potential inhibitors of the MERS-CoV 3CL protease. The Random Forest model, selected for its superior performance, demonstrated robust discriminative power with an AUC-ROC of 0.94, confirming its reliability in distinguishing active from inactive compounds. The confusion matrix further validated the model’s precision, showing 23 correct predictions for class 0 (one false positive) and 21 correct predictions for class 1 (no false negatives), highlighting its high specificity and sensitivity for large-scale screening. Applied to a dataset of 14,194 naturally occurring compounds from PubChem, the model identified 1232 active candidates (35.2%). Among these, Perenniporide B, Terrestrol G, and Phellifuropyranone A exhibited interaction profiles surpassing the reference inhibitor AW4 (CID137348956). The results obtained highlight the strong inhibitory potential of Perenniporide B, Phellifuropyranone A, and Terrestrol G against the MERS-CoV 3CL protease. The molecular interaction analysis revealed that these compounds exploit diverse anchoring mechanisms, combining hydrogen bonds, hydrophobic interactions, and sulfur bonds, which play a key role in stabilization within the enzyme’s active site. Perenniporide B is distinguished by the formation of short and stable hydrogen bonds (1.7–2.9 Å) with critical catalytic residues such as His41 and Asp190, which are directly involved in the proteolytic cleavage mechanism and stabilization of the enzyme’s architecture. Its hydrophobic interaction with Leu144 and Leu49, as well as the moderate sulfur bond with Cys148, suggest the strong affinity and efficient stabilization of the enzyme–inhibitor complex. This combination of interactions gives Perenniporide B optimal structural adaptability to occupy the catalytic pocket and disrupt MERS-CoV enzymatic activity. Terrestrol G, on the other hand, exhibits a diverse interaction profile, involving sulfur bonds with Cys145/Cys148 and several stabilizing hydrogen bonds, notably with Thr26, a key residue in substrate recognition. The ability of Terrestrol G to establish these structural interactions suggests that it can efficiently insert itself into the active site, thus limiting the flexibility required for enzymatic function. In comparison, Phellifuropyranone A also exhibits notable affinity for the 3CL protease, interacting with His41 and Cys148 via sulfur and hydrogen bonds. However, structural analysis suggests that the relative length of these bonds (greater than those observed for Perenniporide B and Terrestrol G) could affect anchor stability. However, the presence of an interaction with Met25 could strengthen its engagement within the active site, contributing to its inhibitory potential. Comparing these compounds to the reference inhibitor AW4, it appears that Perenniporide B and Terrestrol G adopt more specific and stabilizing binding modes, notably due to their ability to target key catalytic residues such as Cys145, His41, and Asp190, which are essential for enzymatic activity. Unlike AW4, which does not interact directly with Cys145 and exhibits lower binding affinity, these new compounds could offer more competitive and effective inhibition. These results highlight the importance of steric and electrostatic complementarity between ligands and the active site to maximize enzymatic inhibition. The presence of polar and aromatic functional groups in the structures of these compounds allows them to interact effectively with the enzymatic cavity, thus reinforcing their stability and inhibitory potential [25,26]. Pharmacokinetically, Terrestrol G and Perenniporide B displayed favorable profiles: acceptable absorption, low drug interaction risks, and promising safety (e.g., Terrestrol G’s high LD_50_ of 2500 mg/kg). However, Perenniporide B exhibited a respiratory toxicity score of 0.669, which, although higher than the other studied compounds, remains within a moderate risk range. Comparatively, Paxlovid (0.991) and Remdesivir (0.963) have been associated with transient respiratory side effects (e.g., dyspnea in 5–10% of Remdesivir recipients) [27], while Molnupiravir (~0.75) has not shown significant clinical respiratory toxicity [28]. Notably, the respiratory toxicity score of Perenniporide B (0.669) is similar to that of Amiodarone (0.658), a drug known to carry a low but notable risk (1–5%) of granulomatous pneumonia [29,30]. These findings indicate that, although Perenniporide B presents a moderate respiratory risk, its toxicity profile remains within an acceptable range for further consideration. However, in vivo validation remains essential to confirm its clinical applicability, as regulatory guidelines (e.g., EMA) recommend further preclinical investigations for compounds with predicted toxicity scores exceeding **0.6**. Since the computational predictions for Perenniporide B fall within this threshold, experimental validation is necessary to accurately assess its potential respiratory effects [31]. Molecular dynamics simulations enabled the evaluation of the stability of the complexes formed. Phellifuropyranone A showed the lowest fluctuations (0.5–4.0 Å), indicating excellent stability, while Perenniporide B and Terrestrol G showed slightly higher variations (0.5–4.5 Å and 0.5–5.4 Å, respectively). In contrast to structural instability, these fluctuations suggest adaptive flexibility, which may promote a better fit to the protease active site and improve enzyme inhibition [32]. Furthermore, the analysis of interaction fraction histograms revealed that AW4 binding was weaker and less stable, while the new inhibitors exhibit more continuous and specific interactions, thus enhancing their antiviral potential. These promising results must, however, be interpreted with caution, as certain methodological limitations remain. In particular, the lack of experimental data on the biological activity of the compounds limits the possibility of establishing a detailed structure–activity relationship (SAR). In our model, the classification of compounds into active and inactive is based on a predictive threshold of 0.1, which is an in silico approach requiring experimental validation to confirm these results. In conclusion, this multidisciplinary strategy accelerates antiviral drug discovery by bridging computational prediction, structural validation, and toxicity assessment. Terrestrol G and Perenniporide B, with balanced efficacy and safety profiles, emerge as prime candidates for MERS-CoV 3CL protease inhibition. Future work should prioritize in vitro and in vivo studies to confirm their therapeutic potential and refine their drug-like properties.

## 4. Materials and Methods

### 4.1. Construction of Predictive Models

To classify compounds based on their antiviral activity against MERS-CoV, five machine learning models were developed: Gradient Boosting (GB) [33], Support Vector Machine (SVM) [34], K-Nearest Neighbors (KNN) [35], Logistic Regression (LR) [36], and Random Forest (RF) [37].

The dataset used for training these models consisted of 78 active compounds (Table S1) retrieved from the ChEMBL database (the relatively small dataset reflects the scarcity of research specifically targeting this protease) and 78 inactive compounds obtained from the PubChem database. The compounds were labeled in a binary manner (1 for active compounds and 0 for inactive ones), with an additional column, “Label”, included to distinguish between the two groups.

Molecular fingerprints, specifically Morgan fingerprints [38], were generated for each compound using RDKit (Version 2022.09.1. https://www.rdkit.org/, accessed 2 January 2025), a widely used cheminformatics toolkit. Morgan fingerprints provide a detailed representation of molecular structures by capturing key chemical features and patterns. These fingerprints served as independent variables in model training, allowing the models to establish relationships between molecular structure and antiviral activity.

**Figure 11:**
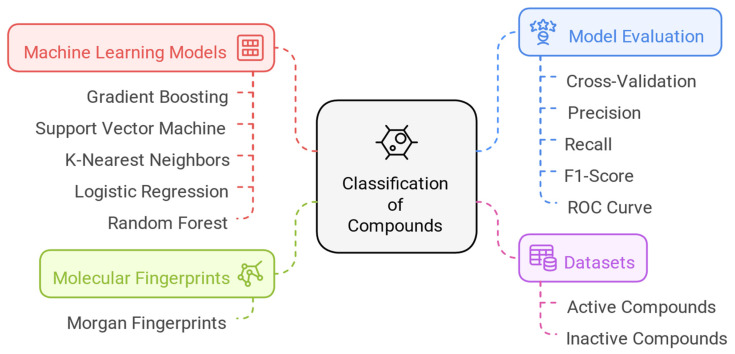
Construction of predictive models.

To evaluate model performance, cross-validation [39] was employed, using key metrics such as precision, recall, F1-score, and the area under the receiver operating characteristic (ROC) curve. Hyperparameter optimization was performed using grid search to enhance predictive performance. After selecting the best-performing model based on validation results, we applied it to a dataset of 14,194 naturally occurring compounds, downloaded from the PubChem database using the search term “natural source”. This screening step identified potential antiviral candidates for further computational analysis. All the steps are summarized in the figure below (Figure 11).

### 4.2. Molecular Docking Simulation

Compounds predicted by our model to be active were subjected to molecular docking simulations to assess their affinity with the target protein. The docking process was performed in several steps.

#### 4.2.1. Ligand Preparation

Compound structures were extracted from the PubChem database (https://pubchem.ncbi.nlm.nih.gov, accessed 10 January 2025). These molecules were prepared for docking by generating 3D conformers and optimizing their geometry using Schrödinger’s LigPrep program, which adjusts hydrogen atoms and determines partial charges.

#### 4.2.2. Receptor Preparation

The structure of the target protein was downloaded from the Protein Data Bank (PDB) under the code 5KWW (https://www.rcsb.org/structure/5KWW. accessed 11 January 2025) [40]. After downloading, water molecules and irrelevant ions were removed using the Protein Preparation Wizard tool in Maestro (Schrödinger LLC 2020-3, New York, NY, USA). Then, hydrogen atoms were added to the structure, taking into account the appropriate proton states for the different functional groups. The protein geometry was optimized by energy minimization to reduce geometric tensions and ensure an optimal energetic conformation. The protein was then prepared for virtual interactions with the target ligands and molecular dynamics simulations.

#### 4.2.3. Active Site Definition

In our study, we first identified the key amino acids of the active site of the MERS-CoV 3CL protease by analyzing the structure of the complex between this protease and the inhibitor GC813 using the tool (https://bio.tools/plip, accessed 18 January 2025). These amino acids were then used to define the dimensions of the docking grid (Glide box) using the Schrödinger software (Schrödinger LLC 2020-3, New York, NY, USA). The dimensions obtained for this active site are as follows: x = −18.83, y = +24.25, and z = +0.94. The grid box size was set to 30 × 30 × 30 Å to ensure sufficient coverage of the binding pocket while maintaining computational efficiency. Finally, our results show that the active site thus defined corresponds to the observations reported in the literature [41], confirming its localization in the cleft between the two domains of the protease and its characterization by the Cys-His catalytic dyad.

#### 4.2.4. Docking Simulation

Molecular docking was performed using Schrödinger’s Glide software (Schrödinger LLC 2020-3, New York, NY, USA), which evaluates the interactions between ligands and the target protein. Glide is a molecular docking program integrated into the Maestro software (Schrödinger LLC 2020-3, New York, NY, USA). designed to predict the optimal position and orientation of ligands in the active site of the target protein. Ligands were docked to the identified active site, and GlideScore (GScore) was used as the primary scoring function, incorporating van der Waals interactions (Lennard–Jones potential), hydrogen bonding, electrostatic interactions, desolvation penalties to account for ligand and receptor hydration states, as well as π-π stacking and salt bridge formation. Compounds with the lowest scores showed the best affinity for the receptor. This docking process allows for the accurate simulation of ligand–protein interactions, and the Discovery Studio software (version 2024) was used to analyze ligand–protein interactions, providing crucial information for the development of new inhibitors.

### 4.3. ADMET Analysis

The three compounds that showed the best affinities in the docking simulations were subjected to an analysis of their ADMET (absorption, distribution, metabolism, excretion, and toxicity) properties to evaluate their pharmacological potential and safety. This analysis was performed using ADMET Lab (https://admetmesh.scbdd.com/, accessed 25 January 2025), which predicted oral bioavailability, cellular permeability, and metabolism and excretion of the compounds. In parallel, the PROTOX program (https://tox.charite.de/protox3/, accessed 25 January 2025) was used to predict the toxicity risks of the compounds, particularly with regard to hepatic, renal, and cardiac toxicity.

### 4.4. Molecular Dynamics (MD) Simulations

Molecular dynamics simulations were carried out using the Desmond software (Schrödinger LLC 2020-3, New York, NY, USA) under the NPT ensemble. The system was maintained at a constant temperature of 300 K and a pressure of 1 bar, with temperature regulation achieved through the Nosé–Hoover thermostat and pressure control via the Martyna–Tuckerman–Klein barostat (coupling constant: 2.0 ps).

The OPLS_2005 force field was employed to model atomic interactions, while long-range electrostatic interactions were computed using the particle mesh Ewald (PME) method with a 9.0 Å cutoff for Coulomb interactions. Water molecules were described using the Simple Point Charge (SPC) model. Short-range non-bonded interactions were updated at every step, whereas long-range interactions were recalculated every three steps. Each simulation spanned 100 ns, with an initial relaxation time of 1 ps.

Trajectory data were collected for subsequent analysis, focusing on four protein–ligand complexes. The stability of these complexes was assessed by examining the Root Mean Square Deviation (RMSD), Root Mean Square Fluctuation (RMSF), and protein–ligand interactions over time. These analyses were conducted using the Simulation Interaction Diagram (SID) tool integrated within Desmond (Schrödinger LLC ***2020-3***, New York, NY, USA).

## 5. Conclusions

In this study, we used machine learning, specifically the Random Forest model, to identify potential inhibitors of the MERS-CoV 3CL protease. The model demonstrated high predictive accuracy, facilitating the selection of natural compounds with high inhibitory potential. Among these, Perenniporide B, Phellifuropyranone A, and Terrestrol G exhibited significant binding energies of −9.17, −9.08, and −8.71 kcal/mol, respectively, indicating efficient interactions with key catalytic residues of the viral enzyme.

Molecular dynamics (MD) simulations over 100 ns confirmed the stability of these compounds within the active site, with RMSD fluctuations less than 2.0 Å for Perenniporide B and Terrestrol G, suggesting stable binding. Furthermore, RMSF analysis revealed reduced flexibility of key interacting residues, including Cys145 and His41, reinforcing the reliability of the observed interactions.

Furthermore, ADMET predictions showed that these compounds possess good pharmacokinetic properties, with high intestinal absorption, low overall toxicity, and acceptable metabolic stability. However, Perenniporide B was identified as presenting a moderate risk of respiratory toxicity, requiring structural optimization to reduce this toxicity while maintaining its strong inhibitory potency.

In future work, we will seek to experimentally validate the efficacy of these compounds through in vitro assays and improve the structure of Perenniporide B to optimize its safety profile. In addition, in-depth studies will be conducted to examine their bioavailability and potential for optimization as promising antiviral agents against MERS-CoV.

## Figures and Tables

**Table 1:** Comparison of performance across multiple models.

	Logistic Regression Model	Support Vector Machine Model	Gradient Boosting Model	K-Nearest Neighbors Model	Random Forest Model
**Accuracy**	0.81	0.78	0.84	0.59	0.97
**ROC-AUC Score**	0.90	0.90	0.93	0.82	0.98
**F1-Score**	0.8	0.77	0.82	0.68	0.98
**Macro Avg**	0.82	0.79	0.85	0.64	0.98
**Weighted Avg**	0.81	0.78	0.84	0.59	0.98

**Table 2:** Top-scoring compounds in docking studies.

Compounds	CID PubChem	Source	XP Score (kcal/mol)
**Perenniporide B**	60199564	Tropicoporus linteus	−9.17
**Phellifuropyranone A**	24770409	Tropicoporus linteus	−9.081
**Terrestrol G**	24761035	Penicillium terrestre	−8.71
**AW4**	137348956	///	−7.34

**Table 3:** Key interactions of selected compounds with MERS-CoV 3CLpro.

Compounds	H-Bond	Distance	Number	Hydrophobic	Distance	Number	Sulfur	Distance
**Perenniporide B**	Asp190 Gln167 Gln192 Glu169 Gly146 Gly146 His41 Leu144 Leu49	2,488 1,944 1,769 2,697 2,915 1,983 2,579 1,709 2,722	9	Cys148 His41 Leu49	5,132 4,569 4,488	3		
**Phellifuropyranone A**	Gly146 His41 His194 Tht29 Thr29 Val193	3,351 3,1879 2,7442 1,7356 2,0224 1,9672	6	Cys148 Cys148 His194 Leu49 Leu49 Met25 Met168	5,150 5,003 5,659 5,117 4,937 4,391 5,491	7	Cys145 Met25	4,583 3,007
**Terrestrol G**	Gln167 Leu49 Lys191 Thr26	1,7358 2,1533 3,7527 1,7978	4	Leu49 Met25	4,876 5,001	2	Cys145 Cys148	5,780 4,357
**AW4**	Cys148 Gln167 Gln192 Gln192 Glu169 Glu169 Leu49 Tyr54 Val193	2.428 2,080 2,405 3,394 1,884 2,723 2,472 2,635 2,674	9	His41 His194 Leu49	4,178 4,549 3,655	3		

**Table 4:** Physicochemical properties of top docking compounds and inhibitor AW4.

Compounds	MW (g/mol)	nRot	nHet	Flexibility	TPSA (Å^2^)	nRing	Log S	Log P
**Perenniporide B**	366.13	7	8	0.538	122.52	2	−3.061	1.554
**Phellifuropyranone A**	378.07	3	7	0.125	124.27	4	−4.103	3.562
**Terrestrol G**	296.05	3	6	0.250	101.15	2	−1.399	2.191
**AW4**	533.16	15	13	0.938	171.13	2	−1.945	1.025

**MW (g/mol)**—molecular weight; should be <500 g/mol (Lipinski’s Rule). **nRot**—number of rotatable bonds; higher values indicate more flexibility. **nHet**—number of heteroatoms; affects hydrogen bonding potential. **Flexibility**—molecular flexibility; influences binding and permeability. **TPSA (Å^2^)**—topological polar surface area; should be <140 Å^2^ for good oral bioavailability. **nRing**—number of rings in the structure; important for drug-like properties. **Log S**—aqueous solubility (log scale); lower values indicate poor solubility. **Log P**—partition coefficient (lipophilicity); should be < 5 (Lipinski’s Rule).

**Table 5:** Drug-likeness and medicinal chemistry properties of top docking compounds and inhibitor AW4.

Compounds	QED	SAscore	Pfizer Rule	Lipinski Rule	Golden Triangle
**Perenniporide B**	0.632	3.953	Accepted	Accepted	Accepted
**Phellifuropyranone A**	0.397	2.808	Accepted	Accepted	Accepted
**Terrestrol G**	0.559	2.716	Accepted	Accepted	Accepted
**AW4**	0.25	3.988	Accepted	Rejected	Rejected

**QED**—quantitative estimate of drug-likeness; higher values indicate better drug-like properties. **SAscore**—synthetic accessibility score; lower values indicate easier synthesis. **Pfizer Rule**—compliance with Pfizer’s drug-likeness criteria; accept/reject. **Lipinski Rule**—compliance with Lipinski’s Rule of Five; accept/reject. **Golden Triangle**—drug-likeness based on lipophilicity and MW; accept/reject.

**Table 6:** Predicted absorption and distribution profiles of top docking compounds and inhibitor AW4.

Compounds	Caco-2 Permeability	HIA%	P-gp Inhibitor	PPB	Vd (L/kg)
**Perenniporide B**	−4.922	65.62	Excellent	80.876	1.259
**Phellifuropyranone A**	−4.985	63.39	Excellent	96.695	0.355
**Terrestrol G**	−5.168	67.33	Excellent	95.148	0.511
**AW4**	−5.607	36.24	Excellent	76.47	0.341

**Caco-2 permeability** estimates intestinal absorption, with more negative values indicating lower permeability. **HIA% predicts human intestinal** absorption, where values above 70% are high. **P-gp inhibitor classification** reflects interaction with P-glycoprotein. **PPB indicates plasma protein binding**, with values above 90% signifying strong binding and lower free drug levels. **Vd (L/kg**) represents drug distribution, where values above 1 L/kg suggest extensive tissue distribution.

**Table 7:** Predicted metabolism and excretion profiles of top docking compounds and inhibitor AW4.

Compounds	CYP1A2 Inhibitor	CYP2C19 Inhibitor	CYP2C9 Inhibitor	CYP2D6 Inhibitor	CYP3A4 Inhibitor	CL (ml/min/Kg)	T1/2 (H)
**Perenniporide B**	0.493	0.04	0.067	0.012	0.059	6.159	0.545
**Phellifuropyranone A**	0.934	0.152	0.447	0.156	0.498	7.691	0.814
**Terrestrol G**	0.418	0.05	0.181	0.444	0.074	13.677	0.966
**AW4**	0.014	0.061	0.166	0.006	0.16	2.121	0.626

**CYP inhibition** values indicate the compounds’ potential to inhibit key cytochrome P450 enzymes, affecting drug metabolism. **CL (clearance)** represents the rate of drug elimination, with higher values indicating faster clearance. **T1/2 (half-life)** reflects the duration a drug remains in the body, where longer half-lives suggest prolonged effects.

**Table 8:** Toxicity prediction using ADMELAB.3.

Compounds	hERG Blockers	AMES Toxicity	Skin Sensitization	Carcinogenicity	Respiratory Toxicity
**Perenniporide B**	0.039	0.347	0.173	0.157	0.669
**Phellifuropyranone A**	0.081	0.036	0.951	0.358	0.106
**Terrestrol G**	0.165	0.524	0.952	0.128	0.134
**AW4**	0.069	0.014	0.062	0.016	0.025

**hERG blockade risk** (potential for cardiotoxicity), **AMES toxicity** (mutagenic potential)**, skin sensitization** (allergic reactions upon skin exposure), **carcinogenicity** (cancer-causing potential), and **respiratory toxicity** (risk of adverse effects on the respiratory system). Lower values generally indicate lower toxicity risks.

**Table 9:** Toxicity prediction using PROTOXII.

Compounds	Hepatotoxicity	Mutagenicity	Cytotoxicity	Ecotoxicity	Ld50
**Perenniporide B**	Inactive	Inactive	Inactive	Inactive	220
**Phellifuropyranone A**	Inactive	Inactive	Inactive	Inactive	800
**Terrestrol G**	Inactive	Inactive	Inactive	Inactive	2500
**AW4**	Inactive	Inactive	Inactive	Inactive	3000

**Hepatotoxicity, mutagenicity, cytotoxicity, and ecotoxicity**, where “inactive” indicates no detected toxicity in these categories. **LD50 values** (lethal dose for 50% of test subjects, measured in mg/kg) suggest acute toxicity levels, with higher values indicating lower toxicity.

## Data Availability

Data are contained within the article.

## Supplementary Materials

The following supporting information can be downloaded at: https://www.mdpi.com/article/10.3390/ijms26073047/s1.
