# Supplementary material for: Machine Learning-Guided Screening and Molecular Docking for Proposing Naturally Derived Drug Candidates Against MERS-CoV 3CL Protease
Source: Int J Mol Sci. 2025 Mar 26;26(7):3047. doi: 10.3390/ijms26073047 (PMC11988297; doi:10.3390/ijms26073047)
Supplement: Supplementary file 1 [file ijms-26-03047-s001.zip › ijms-3527250-supplementary.pdf]

Machine Learning-Guided Screening and Molecular Docking for Proposing Naturally Derived Drug Candidates Against MERS-CoV 3CL Protease

Mebarka Ouassaf[1], Radhia Mazri[1], Shaf Ullah Khan[2,3] Kannan R. R. Rengasamy[4,5] Bader Y. Alhatlani[6]

[1] Group of Computational and Medicinal Chemistry, LMCE Laboratory, University of Biskra, Biskra, Algeria;

[2] UNICAEN, Inserm U1086 ANTICIPE (Interdisciplinary Research Unit for Cancer Prevention and Treatment), Normandie Univ, Caen, France;

[3] Cancer Centre François Baclesse, UNICANCER, Caen, France ;

[4] Laboratory of Natural Products and Medicinal Chemistry (LNPMC), Department of Pharmacology, Saveetha Dental College and Hospitals, Saveetha Institute of Medical and Technical Sciences (SIMATS), Thandalam, Chennai, India;

[5] Centre of Excellence for Pharmaceutical Sciences, North-West University, Potchefstroom, 2520, South Africa;

[6] Unit of Scientific Research, Applied College, Qassim University, Buraydah 52571, Saudi Arabia; balhatlani@qu.edu.sa (B.Y.A.)

* Correspondence: balhatlani@qu.edu.sa (B.Y.A.); nouassaf@univ-biskra.dz (M.O.)

Table s1: *List of Active Compounds Used in the Study*

Molecule	Smiles
CHEMBL510038	Nc1nnc(Sc2ncc([N+](=O)[O-])s2)s1
CHEMBL4303312	Oc1c(I)cc(I)c(O)c1I
CHEMBL4303622	N#CSc1cc(SC#N)c(N)nc1N
CHEMBL1591074	c1ccc2sc(SNC3CCCCC3)nc2c1
CHEMBL1382627	Nc1ccc(S(=O)(=O)[N-]c2ncccn2)cc1.[Ag+]
CHEMBL297453	O=C(O[C@@H]1Cc2c(O)cc(O)cc2O[C@@H]1c1cc(O)c(O)c(O)c1)c1cc(O)c(O)c(O)c1
CHEMBL8260	O=c1cc(-c2ccccc2)oc2cc(O)c(O)c(O)c12
CHEMBL1255778	Cl.NC(CO)C(=O)NNCc1ccc(O)c(O)c1O
CHEMBL329522	O=C(c1cc(O)c(O)c(O)c1)c1ccc(O)c(O)c1O
CHEMBL406050	CCC(C)SSc1ncc[nH]1
CHEMBL596674	CC(C)N(CCC(=O)c1ccc2ccccc2c1)Cc1ccccc1
CHEMBL477197	Oc1cc2c(cc1O)[C@@H]1c3ccc(O)c(O)c3OC[C@]1(O)C2
CHEMBL4212620	CCC1(OC(=O)N[C@@H](CC(C)C)C(=O)N[C@H](C=O)C[C@@H]2CCNC2=O)CCN(C(=O)OC(C)(C)C)CC1
CHEMBL4214066	CC(C)C[C@H](NC(=O)OC1CCN(C(=O)OC(C)(C)C)CC1)C(=O)N[C@H](C=O)C[C@@H]1CCNC1=O
CHEMBL4475926	Cc1cc(C)n(S(=O)(=O)c2ccc(N)cc2)c(=O)n1
CHEMBL4303376	Cc1ccccc1N(C)C(=O)c1ccc([S+]([O-])c2nccs2)c([N+](=O)[O-])c1
CHEMBL4303595	O=C1C=Cc2cc(Br)ccc2C1=O
CHEMBL4217568	CCOC(=O)N1CCC(OC(=O)N[C@@H](CC(C)C)C(=O)N[C@H](C=O)C[C@@H]2CCNC2=O)CC1
CHEMBL3348861	O=C1c2c(O)cc(O)cc2O[C@H](c2cc(O)c(O)c(O)c2)[C@H]1O
CHEMBL1256915	CN1CC[C@@]2(C)c3cc(O)ccc3N(C)[C@@H]12.O=C(O)/C=C/C(=O)O
CHEMBL1474701	O=C1C=CC(=O)C2=C1C1c3ccccc3C2c2ccccc21
CHEMBL518800	CC[C@H](CO)Nc1nc(NCc2ccc(-c3ccccn3)cc2)c2ncn(C(C)C)c2n1
CHEMBL571700	CN(C)C(=S)SC(=S)N(C)C
CHEMBL465704	Nc1ccc2cc3ccc(N)cc3nc2c1.Nc1ccc2cc3ccc(N)cc3nc2c1.O=S(=O)(O)O

CHEMBL1161936	O=C(O)c1ccc(SSc2ccc(C(=O)O)cn2)nc1
CHEMBL1886408	CCOC(=O)Cc1ccc(-c2ccccc2)cc1
CHEMBL128251	O=C(O)CCCCCCCCCCCCCc1ccc(I)cc1
CHEMBL4303361	Oc1cc(CNC(=S)/C=C/c2cc(O)c(O)c(Br)c2)cc(O)c1O
CHEMBL4209146	CC(C)C[C@H](NC(=O)OC1CCN(S(C)(=O)=O)CC1)C(=O)N[C@H](C=O)C[C@@H]1CCNC1=O
CHEMBL472940	Oc1ccc2ccccc2c1SSc1c(O)ccc2ccccc12
CHEMBL165012	O=[N+]([O-])c1ccc(N2CCNCC2)cc1
CHEMBL4303595	O=C1C=Cc2cc(Br)ccc2C1=O
CHEMBL164	O=c1c(O)c(-c2cc(O)c(O)c(O)c2)oc2cc(O)cc(O)c12
CHEMBL508280	O=[N+]([O-])c1cnc(Sc2nnc(O)n2-c2ccc3c(c2)OCCO3)s1
CHEMBL11350	O=C(NC1=CC=CC2C(=O)C=C(c3nn[nH]n3)OC12)c1ccc(OCCCCc2ccccc2)cc1
CHEMBL444186	CC(CN1CC(=O)NC(=O)C1)N1CC(=O)NC(=O)C1
CHEMBL1380480	COc1cc(C(=S)N2CCOCC2)ccc1O
CHEMBL1408862	O=[N+]([O-])C(Br)(CO)CO
CHEMBL1200930	COCCCOc1ccnc(C[S+]([O-])c2nc3ccccc3[n-]2)c1C.[Na+]
CHEMBL1256832	Cl.Oc1cc2c(cc1O)[C@H]1c3ccccc3CN[C@@H]1CC2
CHEMBL2354521	COc1cccc(C(=O)CCN[C@@H](C)[C@H](O)c2ccccc2)c1.Cl
CHEMBL1200471	[O-][n+]1ccccc1[S-].[O-][n+]1ccccc1[S-].[Zn+2]
CHEMBL1743207	O=[N+]([O-])c1ccc(Cl)c2nonc12
CHEMBL480	Cc1c(OCC(F)(F)F)ccnc1C[S+]([O-])c1nc2ccccc2[nH]1
CHEMBL388676	O=C1C2CC=CCC2C(=O)N1SC(Cl)(Cl)Cl
CHEMBL51085	O=c1c2ccccc2[se]n1-c1ccccc1
CHEMBL964	CCN(CC)C(=S)SSC(=S)N(CC)CC
CHEMBL1271993	O=C1C(Cl)=C(N2CCOCC2)C(=O)N1c1ccc(Cl)c(Cl)c1
CHEMBL3545157	O=c1sn(-c2cccc3ccccc23)c(=O)n1Cc1ccccc1
CHEMBL120563	CN(C)C(=S)SSC(=S)N(C)C
CHEMBL9116	CNC(=O)ON(C(C)=O)C(=O)NC
CHEMBL178459	Cc1c(-c2cnccn2)ssc1=S
CHEMBL313154	Nc1cc2c3ccccc3ccc2c2ccccc12

CHEMBL69863	Oc1cc(O)cc(/C=C/c2ccc(O)c(O)c2)c1
CHEMBL297453	O=C(O[C@@H]1Cc2c(O)cc(O)cc2O[C@@H]1c1cc(O)c(O)c(O)c1)c1cc(O)c(O)c(O)c1
CHEMBL3545085	CC(C)c1cc(CNc2nc(Nc3cc(C4CC4)[nH]n3)cc(N3CCN(C)CC3)n2)on1
CHEMBL1475252	COc1ccc2[nH]c([S+]([O-])Cc3ncc(C)c(OC)c3C)nc2n1
CHEMBL3545157	O=c1sn(-c2cccc3ccccc23)c(=O)n1Cc1ccccc1
CHEMBL505308	Br.Oc1cc2c(cc1O)C(c1ccccc1)CNCC2
CHEMBL52	CC(Cc1ccc(O)c(O)c1)C(C)Cc1ccc(O)c(O)c1
CHEMBL28	O=c1cc(-c2ccc(O)cc2)oc2cc(O)cc(O)c12
CHEMBL178459	Cc1c(-c2cnccn2)ssc1=S
CHEMBL1096	CC(C)c1ccc2oc3nc(N)c(C(=O)O)cc3c(=O)c2c1
CHEMBL271023	[O-][S+](c1ccccc1)c1ccc2nnnn2n1
CHEMBL1201236	C[C@@](Cc1ccc(O)c(O)c1)(NN)C(=O)O
CHEMBL324842	O=C(/C=C/c1ccc(O)c(O)c1)O[C@H](Cc1ccc(O)c(O)c1)C(=O)O
CHEMBL1908397	O=C(c1ccc(/C=C/c2n[nH]c3ccccc23)cc1)N1CCNCC1
CHEMBL488755	CN(C)CCNc1nc2cc(O)ccc2c2c1C(=O)c1ccccc1-2
CHEMBL1917204	Cc1ccc(-n2sc(=O)n(Cc3ccc(F)cc3)c2=O)cc1
CHEMBL270299	CC(C)(C)c1ccc(S(=O)(=O)/C=C/C#N)cc1
CHEMBL490129	C[n+]1cc2c3c(ccc2c2ccc4cc5c(cc4c21)OCO5)OCO3.[Cl-]
CHEMBL55400	Nc1ccc2cc3ccc(N)cc3nc2c1
CHEMBL964	CCN(CC)C(=S)SSC(=S)N(CC)CC
CHEMBL403183	Cc1ccc(S(=O)(=O)/C=C/C#N)cc1

Table s2 *List of Compounds with XP Scores Better than the Reference Compound*

smile	Score xp kcal/mol

COC(=O)c1ccc(O)c(NC(=O)CC[C@]2(C)C(=O)C=C[C@@]34C[C@@H](CC[C@@H]32)[C@](O)(CO)C4)c1O	-9.70329
CCc1c(OC)cc2c(c1O)C(=O)C=C(OC)[C@@]2(O)C[C@@H](O)C(=O)OC	-9.18022
C[C@@H](O)c1c(O)cc2c(c1O)C(=O)c1c(O)cc(O)cc1C2=O	-9.17765
O=c1oc(/C=C/c2ccc(O)c(O)c2)cc2oc(-c3ccc(O)c(O)c3)cc12	-9.08114
CC[C@H]([NH3+])[C@H](O)[C@H](O)[C@H](C)C(=O)N[C@@H](CC(C)C)[C@@H]1Cc2cccc(O)c2C(=O)O1	-9.07712
C=C[C@H](Cl)[C@@](C)(O)c1cc(=O)c2c(CO)cc3c(c2o1)C(=O)c1c(O)cccc1C3=O	-8.90016
O=C1[C@@H](O)c2c(O)cc3c(c2[C@H]2O[C@@H]12)-c1ccc(O)c2c(O)ccc-3c12	-8.83441
O=C1c2c(O)cc(O)cc2O[C@H](c2ccc(O)c(O)c2)[C@H]1O	-8.83092
OCc1cc(O)c(Cc2cc(O)ccc2O)c(Cl)c1O	-8.78228
C=C[C@@H](Cl)[C@@](C)(O)c1cc(=O)c2c(CO)cc3c(c2o1)C(=O)c1c(O)cccc1C3=O	-8.7409
c1c(O)ccc(O)c1Cc2c(Cl)c(O)c(CO)cc2O	-8.7124
O=c1c(O)c(-c2ccc(O)cc2)oc2c(O)c(O)cc(O)c12	-8.68966
O=c1c(O)c(-c2cc(O)c(O)c(O)c2)oc2cc(O)ccc12	-8.60532
COc1cc(O)c2c(O)c3c(cc2c1)C[C@@H](C[C@H](O)C[C@@H](O)CCCCCO)OC3=O	-8.58004
CC(C)C[C@H](NC(=O)[C@@H](O)[C@@H](O)[C@H]1CCC[NH2+]1)[C@H]1Cc2cccc(O)c2C(=O)O1	-8.52604
O=C1c2c(O)cc(O)cc2O[C@H](c2cc(O)c(O)c(O)c2)[C@H]1O	-8.5198
COc1cc(O)c2c3c(c(O)c(O)c(C)c13)C(=O)O[C@@H]2O	-8.49538
COc1cc(O)c2c(O)c3c(cc2c1)C[C@@H](C[C@@H](O)C[C@@H](O)CCCCCO)OC3=O	-8.49304
O=c1c(O)c(-c2ccc(O)c(O)c2)oc2cc(O)c(O)c(O)c12	-8.34837
O=c1c(O)c(-c2ccc(O)cc2O)oc2cc(O)cc(O)c12	-8.32984
COC(=O)[C@]1(O)C(c2ccc(O)cc2)=C(O)C(=O)[C@@H]1c1ccc(O)cc1	-8.27322
COc1cc(-c2oc3cc(O)cc(O)c3c(=O)c2O)ccc1O	-8.26345
Oc1ccc2c(c1)OC[C@]1(O)Cc3cc(O)c(O)cc3[C@H]21	-8.24084
Cc1c(O)c(O)c(O)c2c1CO[C@@]1(OCc3c(C)c(O)c(O)c(O)c31)C2=O	-8.22426
C[C@@H](O)Cc1cc(O)c2c(c1)C(=O)c1c(O)c(O)cc(O)c1C2=O	-8.19576
O=c1c(-c2ccc(O)c(O)c2)coc2c(O)c(O)cc(O)c12	-8.18112
O=c1c(O)c(-c2ccc(O)c(O)c2)oc2cc(O)cc(O)c12	-8.12489
CC[C@H]1C=CC(=O)O[C@H]1/C=C/[C@@](C)(O)[C@H](O)C[C@@H](O)/C=C\C=C/[C@@H]1CCC[C@H](O)C1	-8.12313
COc1cc(O)c2c(=O)oc3c(O)c(OC)cc(O)c3c2c1	-8.10808

COc1cc(O)c2c(O)c3c(cc2c1)C[C@@H](C[C@H](O)C[C@H](O)C[C@H](C)O)OC3=O	-8.0625
CC[C@@H](C)C[C@@H](C)/C=C/[C@@H]1C=C[C@@H](O)[C@H](O)[C@]12C(=O)O[C@](C)(O)[C@@H]2O	-8.06152
O=c1c(O)c(-c2ccc(O)c(O)c2)oc2cc(O)ccc12	-8.05561
CC(C)=CCc1c(O)c(O)c(O)c2c1CC(=O)O[C@@H]2C	-8.03348
COc1cc(O)c2c(O)c3c(cc2c1)C[C@@H](C[C@@H](O)C[C@H](O)CCCCCO)OC3=O	-8.00937
C[NH2+][C@H](C(=O)N(C)[C@@H](Cc1ccccc1)C(=O)NCCc1ccccc1)C(C)C	-8.00805
COc1cc(O)c2c(O)c3c(cc2c1)C(=O)C(C)=CC3=O	-8.00707
CC/C=C\C=C/C(=O)N/C=C/C[C@H]1C[C@H]2C[C@H](O)C[C@H](Cc3cccc(O)c3C(=O)O1)O2	-8.00398
COc1cc(O)c2c(c1)/C=C\C[C@H](O)[C@H](O)[C@H](O)/C=C\C[C@H](C)OC2=O	-7.96588
Oc1ccc(O)c2c1[C@@H](O)[C@H]1O[C@H]1[C@]21Oc2cccc3c(O)ccc(c23)O1	-7.94534
COc1cc(O)c2c(=O)oc3c(O)c(OC)cc(CO)c3c2c1	-7.9424
COc1cc2oc(-c3ccc(O)c(O)c3)c(OC)c(=O)c2c(O)c1OC	-7.92474
O=c1c(O)c(-c2ccc(O)cc2)oc2cc(O)cc(O)c12	-7.88844
C/C(=C\Cc1cc(C(=O)[O-])ccc1O)CC/C=C(\C)CC[C@H](O)[C@@]1(C)CCC(=O)O1	-7.87943
CC(=O)N[C@@H](CC(C)C)C(=O)N(C)[C@@H](Cc1ccccc1)C(=O)N/C=C/c1c[nH]c2ccccc12	-7.8521
COc1c(O)cc(O)c2c(=O)c(-c3ccc(O)c(O)c3)coc12	-7.84334
COc1cc(O)c2c(O)c3c(cc2c1)C[C@@H](C[C@H](O)C[C@H](O)CCCCCO)OC3=O	-7.83167
CC(=O)C[C@H]1Cc2cc3c(c(O)c2C(=O)O1)C(=O)c1c(O)cccc1C3=O	-7.81712
COC1=CC(=O)c2c(O)c3c(c(O)c2C1=O)[C@H](O)[C@H](C)OC3=O	-7.8099
COc1cc(O)c2c(=O)c3c(O)ccc(O)c3oc2c1	-7.80874
C[C@]1(O)CC(=O)c2c(cc(O)c3c(O)cc(O)cc23)O1	-7.73352
C[C@H](/C=C/C(=O)[O-])[C@H](O)[C@H](C)C[C@H](C)[C@H](O)CC(=O)c1ccc(N)cc1	-7.73332
O=c1oc2cc3cc(O)c(O)cc3oc-2c1-c1ccccc1	-7.71726
CCCc1cc(O)cc2c1C(=O)c1c(O)ccc(O)c1C2=O	-7.69003
Cc1cc(O)c2c(c1O)C(=O)c1cccc(O)c1C2=O	-7.68826
C[C@@]1(O)CC(=O)c2c(cc(O)c3c(O)cc(O)cc23)O1	-7.68825
COC1=CC(=O)c2c(O)c3c(c(O)c2C1=O)C[C@](C)(O)C[C@@H]3O	-7.68495
COc1cc(O)c2c(=O)oc3cc(O)c(O)cc3c2c1	-7.67692
C[C@](O)(CC(N)=O)Cc1cc(O)c2c(c1O)C(=O)c1cccc(O)c1C2=O	-7.67587

COn1cc(-c2ccccc2)c(=O)c([C@@H]2O[C@H]([C@H](C)CCOC(C)=O)[C@@H](C)C[C@H]2C)c1O	-7.66885
O=C1C[C@@H](c2ccc(O)c(O)c2)Oc2cc(O)cc(O)c21	-7.66023
CCc1[nH]c(=O)c(C(C)C)nc1C(=O)N[C@H](CC(C)C)C(=O)N[C@@H](C)C(=O)O	-7.64007
CC(C)=CCc1ccc(O)c(C=O)c1/C=C/[C@@H](O)[C@@H](C)O	-7.63499
COc1c(-c2ccc(O)c(O)c2)oc2cc(O)cc(O)c2c1=O	-7.6251
CCO[C@H]1Cc2c(O)ccc3c2C(=C2c4cccc(O)c4C(=O)C[C@H]23)[C@H]1O	-7.62299
COc1cc(O)c2c(c1)[C@H]1C[C@@H](O)[C@H](O)C[C@]1(C)OC2=O	-7.62108
C[NH2+][C@H](C(=O)N(C)[C@@H](Cc1ccccc1)C(=O)NCCc1c[nH]c2ccccc12)C(C)C	-7.60301
CC(=O)c1cc2cc(CCc3cc4c(o3)C(=O)c3ccccc3C4=O)c(O)c(O)c2c(=O)o1	-7.57503
C[C@H](O)[C@H]1C=Cc2cccc3c2[C@@H](C[C@](C)(O)O3)O1	-7.5722
CCCCCCC[C@]1(O)Cc2c(O)c3c(c(O)c2CO1)C(=O)C(=O)C(OC)=C3	-7.56926
COc1cc(O)c2c(O)c3c(cc2c1)C[C@@H](C[C@H](O)C[C@H](O)C[C@@H](C)O)OC3=O	-7.56537
COc1c(O)cc2oc(-c3ccc(O)c(O)c3)cc(=O)c2c1O	-7.51995
COc1cc([C@@H](Cc2ccccc2)/N=C2\C=C(O)c3c(O)cc(O)cc3C2=O)oc(=O)c1	-7.51
COC1=CC(=O)c2c(O)c(C)c(C[C@H](C)O)c(O)c2C1=O	-7.50039
COc1cc(OC)c2c(O)c3c(cc2c1)[C@H](O)[C@H](C)OC3	-7.4833
Cc1cc(O)c2oc3c(O)cccc3c(=O)c2c1O	-7.47673
CC(C)C[C@H](NC(=O)[C@@H](O)[C@@H]1C=CC(=O)O1)[C@@H]1Cc2cccc(O)c2C(=O)O1	-7.47611
O=c1c2c(O)cc(O)cc2oc2cc(CO)cc(CO)c12	-7.46975
COc1c(O)ccc(-c2coc3cc(O)cc(O)c3c2=O)c1O	-7.43536
COc1cc(O)c2c(O)cc(C(=O)[C@H](C)O)cc2c1	-7.43141
COC1=CC(=O)c2c(O)c3c(c(O)c2C1=O)[C@@H](O)[C@H](C)OC3=O	-7.42619
COC1=C[C@H](O)[C@H](C/C=C(/C)CC/C=C(/C)C[C@H](O)/C=C(/C)CO)[C@@H](C)C1=O	-7.42241
C/C(=C\CC[C@@H]1C[C@H](c2cc(O)ccc2O)OC1=O)CCC=C(CO)CO	-7.41985
COc1cc(O)c2c(=O)c(O)c(-c3ccc(O)c(O)c3)oc2c1	-7.41013
COC(=O)[C@]1(Cc2ccc(O)c(C[C@@H](O)C(C)(C)O)c2)OC(=O)C(OC)=C1c1ccc(O)cc1	-7.39515
COc1cc(OC)c2c(O)c3c(cc2c1)[C@H](O)[C@@H](C)OC3	-7.39447
C[C@H](O)Cc1cc(O)c2c(c1)C(=O)c1cc(O)cc(O)c1C2=O	-7.38325
COC1=CC(=O)c2c(O)c3c(c(O)c2C1=O)[C@H](OC)[C@H](C)OC3=O	-7.37097

COc1cc(OC)c2c(c1)cc(O)c1c(=O)cc(C)oc12	-7.36615
O=C1C[C@@H](O)[C@]2(Oc3cccc4c(O)ccc(c34)O2)c2c(O)ccc(O)c21	-7.36566
O=C1C[C@H]2CC(=O)c3c(O)ccc4c3[C@H]2C(=C2C=CC(=O)c3c(O)ccc-4c32)c2cccc(O)c21	-7.35825
O=c1cc(-c2ccc(O)c(O)c2)oc2cc(O)cc(O)c12	-7.35209
O=c1cc(-c2ccccc2)c2cc(O)c(O)cc2o1	-7.35048
O=C1C[C@@H](c2ccc(O)c(O)c2)Oc2cc(O)ccc21	-7.34647

Table s3: *Clinically Approved and Investigational Compounds for Respiratory Toxicity Comparison*

Compound	Class/Use	Reported/Predicted Respiratory Toxicity
**Paxlovid**	COVID-19 antiviral (protease inhibitor)	- **Score: 0.991** (ADMELab)<br>- Clinical: Transient dysgeusia, no severe respiratory toxicity reported.
**Remdesivir**	COVID-19 antiviral (RNA polymerase inhibitor)	- **Score: 0.963** (ADMELab<br>- Adverse effects: Dyspnea (5-10% of patients).
**Molnupiravir**	COVID-19 antiviral (mutagenic)	- **Score: ~0.75** (ADMELab)<br>- No significant respiratory toxicity reported clinically.
**Amiodarone**	Antiarrhythmic	- **Score: 0.658** (ADMELab)<br>- Clinical: Granulomatous pneumonia (1–5%).

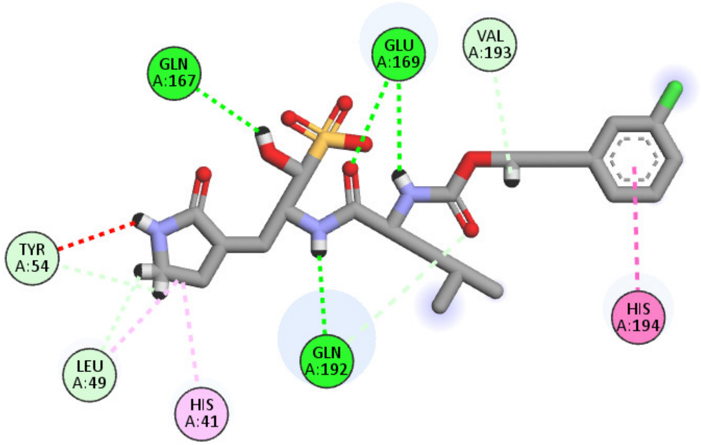

Figure S1 *Molecular Interaction Analysis of the MERS-CoV 3CLpro Complex with AW4*
